# One thousand DNA barcodes of piranhas and pacus reveal geographic structure and unrecognised diversity in the Amazon

**DOI:** 10.1038/s41598-018-26550-x

**Published:** 2018-05-30

**Authors:** Valeria N. Machado, Rupert A. Collins, Rafaela P. Ota, Marcelo C. Andrade, Izeni P. Farias, Tomas Hrbek

**Affiliations:** 1 Laboratório de Evolução e Genétic Animal, Departamento de Genética, Universidade Federal do Amazonas, Av., General Rodrigo Otávio Jordão, 3000, 69077-000 Manaus, AM Brazil; 2 School of Biological Sciences, University of Bristol, Life Sciences Building, 24 Tyndall Avenue, Bristol, BS8 1TQ UK; 3 Programa de Pós-Graduação em Biologia de Água Doce e Pesca Interior, Instituto Nacional de Pesquisas da Amazônia, Av. André Araújo, 2936, CP 2223, Petrópolis, 69080-971 Manaus, AM Brazil; 4 Programa de Pós-Graduação em Ecologia Aquática e Pesca, Instituto de Ciências Biológicas, Universidade Federal do Pará, Av. Perimetral, 2651, Terra Firme, 66040-830 Belém, PA Brazil

## Abstract

Piranhas and pacus (Characiformes: Serrasalmidae) are a charismatic but understudied family of Neotropical fishes. Here, we analyse a DNA barcode dataset comprising 1,122 specimens, 69 species, 16 genera, 208 localities, and 34 major river drainages in order to make an inventory of diversity and to highlight taxa and biogeographic areas worthy of further sampling effort and conservation protection. Using four methods of species discovery—incorporating both tree and distance based techniques—we report between 76 and 99 species-like clusters, i.e. between 20% and 33% of *a priori* identified taxonomic species were represented by more than one mtDNA lineage. There was a high degree of congruence between clusters, with 60% supported by three or four methods. Pacus of the genus *Myloplus* exhibited the most intraspecific variation, with six of the 13 species sampled found to have multiple lineages. Conversely, piranhas of the *Serrasalmus rhombeus* group proved difficult to delimit with these methods due to genetic similarity and polyphyly. Overall, our results recognise substantially underestimated diversity in the serrasalmids, and emphasise the Guiana and Brazilian Shield rivers as biogeographically important areas with multiple cases of across-shield and within-shield diversifications. We additionally highlight the distinctiveness and complex phylogeographic history of rheophilic taxa in particular, and suggest multiple colonisations of these habitats by different serrasalmid lineages.

## Introduction

Piranhas are one of the iconic animals of the Amazon, and as vividly expressed by Theodore Roosevelt^1^ they are often demonised in popular culture as voracious man-eaters: “The head with its short muzzle, staring malignant eyes, and gaping, cruelly armed jaws, is the embodiment of evil ferocity”. Yet despite their infamy, piranhas are poorly known in terms of species diversity, and in common with Amazon ichthyofauna in general are insufficiently investigated taxonomically^2^.

Together with the mostly herbivorous pacus and silver dollars, piranhas comprise the family Serrasalmidae (Ostariophysi: Characiformes), currently with 94 valid species in 16 genera^3^. Endemic to the Neotropics, extant serrasalmids are native to all major drainages east of the Andes including the Amazon, Orinoco, São Francisco, Essequibo (plus smaller coastal Atlantic rivers), Paraná-Paraguay, and also the Maracaibo basin^4^. While some species such as the tambaqui (*Colossoma macropomum*) and the piranhas *Serrasalmus rhombeus* and *Catoprion mento* are widely distributed and found in most major river systems^4^, others have highly restricted distributions. *Ossubtus xinguense*, for example, is endemic to the rapids of the middle Xingu River^5^, while *Mylesinus paucisquamatus* is endemic to the rapids of the Tocantins River^4,6,7^. Other rheophilic species of the genera *Tometes* and *Utiaritichthys* are also endemic to the rapids of other rivers^8–10^. Important commercial fisheries, subsistence fisheries, and aquaculture industries exist for *Colossoma macropomum*, *Piaractus* spp., and *Mylossoma* spp.^11,12^. Serrasalmids are a morphologically, ecologically, and behaviourally diverse group with a variety of feeding strategies and associated morphological adaptations^13–15^; *Colossoma* and *Piaractus* are fruit and seed eaters inhabiting large rivers and are important dispersers of seeds in flooded forests^13,16^; *Tometes*, *Mylesinus*, *Utiaritichthys* and *Ossubtus* are rheophiles and specialist consumers of aquatic plants (Podostemaceae) growing in rapids^5^; while *Acnodon normani* and *Catoprion mento* specialise in eating the scales of other fishes^17,18^.

Morphologically the family is characterised by a deep and compressed body, pre-dorsal spine, and abdominal and pelvic spines forming a ventral keel^4,19^. While the 38 species of piscivorous true piranhas of the genera *Serrasalmus*, *Pygocentrus*, *Pygopristis* and *Pristobrycon* can be easily diagnosed by their single row of sharp interlocking teeth, the remaining genera–a non-monophyletic group of 56 species commonly known as “pacus” (e.g. *Myleus*, *Myloplus*, *Piaractus*) or “silver dollars” (*Metynnis*)–possess two rows of molariform or incisiform premaxillary teeth^5,8,19–22^. Phylogenetic hypotheses based either on morphological^23,24^ or molecular^25,26^ characters have been published. Yet despite these phylogenetic studies and an investigation into broad biogeographic patterns^27^, only limited effort has been applied to understanding intraspecific variation, save for a limited number of phylogeographic studies of species of commercially important genera such as *Colossoma*^28^, *Piaractus*^29^ and *Mylossoma*^30^. Broad coverage DNA barcoding studies of ichthyofauna including serrasalmids are also lacking in the Neotropics, with the exception of the Paraná River basin^31,32^.

Molecular techniques are being increasingly used as a tool for biodiversity inventories^33,34^, and are particularly useful for quickly characterising hyperdiverse tropical communities and flagging new candidate species^31^. Although single locus methods such as DNA barcoding provide insufficient evidence for the description of an individual species without supporting data^35–38^, when viewed in the broader contexts of either higher taxonomic rank or geographic area, DNA-barcode species estimates have been shown to be congruent with traditional taxonomy^39–41^. Methods generating these species-like clusters of sequences–also known as candidate species, OTUs, MOTUs, or mtDNA lineages–are best described in terms of “species discovery” rather than “species delimitation”, as the latter requires multiple character sets^42^; thus, we refer to them as single-locus species-discovery (SLSD) methods. Despite the limitations of inferences based on a single locus, conceptual advances incorporating coalescent theory^33,43–45^ have improved the theoretical justification of tree-based genealogical methods over simpler distance methods requiring arbitrary or generalised distance thresholds^42^. Importantly, by using multiple methods it is possible to counter potential biases and the lack of statistical power associated with any individual method^46,47^, while the provision of confidence intervals enables researchers to capture the genealogical and phylogenetic uncertainty inherent in species delimitation^48,49^.

Given the lack of a geographically wide-ranging DNA-based assessment of serrasalmids, we aim to provide the first detailed inventory of this group with the ultimate aim of better guiding conservation priorities and highlighting groups in need of taxonomic revisions. Specifically, we will: (1) generate a DNA barcode dataset for serrasalmids from the Brazilian Amazon, but also include data from the Orinoco, São Francisco and Paraná river basins where available; (2) make an overall inventory of molecular variation using SLSD methods; (3) identify taxa and geographic areas harbouring previously unrecognised lineages (with particular reference to rheophilic groups).

## Methods

### Sample collection

Muscle and fin-membrane tissue samples were taken in the field from the right-hand pectoral-fin base or from the right side of the flank, and were stored in 95% ethanol and deposited in the tissue collection of the Laboratόrio de Evolução e Genética Animal (LEGAL) at the Universidade Federal do Amazonas (UFAM). Voucher specimens were fixed in 10% formalin and deposited in the fish collection of the Instituto Nacional de Pesquisas da Amazônia (INPA). Vouchers were identified to species by taxonomic specialists using available comparative material, identification keys, original descriptions, and redescriptions of species^8,50–54^. Individuals that could not be identified to species level were reported as “*Genus* sp.” (possible new/unidentified species) or as “*Genus* aff. *species*” (closely related species, possibly new).

Field collections in Brazil were authorised by IBAMA/MMA 045/2008-2011, IBAMA/SISBIO 11325-1, and access to genetic resources was authorised by permit No. 034/2005/IBAMA. IBAMA field collection permits are conditional that collection of organisms be undertaken in accordance with the ethical recommendations of the Conselho Federal de Biologia (CFBio; Federal Council of Biologists), Resolution 301 (December 8, 2012). Field collections in Colombia were authorised by the “Permiso Marco de Investigaciόn” granted by the Ministerio de Ambiente y Desarrollo Sostenible to the Universidad de los Andes, Bogota, Colombia. Collections in French Guiana were authorised under permit APA-973-7 for collections in the core area of the French Guiana Amazonian Park.

### DNA barcode sequence generation

Total DNA was isolated from approximately 50 mg of tissue using standard phenol-chloroform extraction methods^55^. A fragment of 651/657 bp of mitochondrial cytochrome *c* oxidase subunit I (COI) was amplified using the M13-tailed primer cocktails FishF2/FishR2 and VF2/VR1d respectively^56^. The 15 *μ*L PCR mix included 1.2 *μ*L of 10 mM dNTPs (2.5 mM each DNTP), 1.5 *μ*L 10× buffer (75 mM Tris HCL, 50 mM KCL, 20 mM (NH_4_)_2_SO_4_), 1.2 *μ*L 25 mM MgCl_2_, 1.5 *μ*L of primer cocktails (2 pmol each), 0.5 *μ*L of Taq DNA polymerase, 1 *μ*L of template DNA and 6.6 *μ*L ddH_2_O. PCR conditions were: 94 °C (30 sec); 35 cycles of 94 °C (30 sec), 50 °C (35 sec), 72 °C (90 sec); followed by 72 °C (5 min). Amplicons obtained were purified and then sequenced bidirectionally on an automatic ABI 3500 sequencer (Applied Biosystems).

The forward and reverse chromatograms were assembled into contigs using Geneious 7.0.6^57^ and edited manually where required. The sequences were then aligned using Mafft v7.307^58^, and checked manually for insertions, deletions or stop codons using translated amino acids in Geneious. The alignment was trimmed to 621 bp to reduce missing data and erroneous base calls at the ends of the contigs^59^.

Further sequences from the GenBank database were added to the dataset. Using the rentrez_1.0.4 interface^60^ we searched GenBank in July 2017 using the terms “Serrasalmidae” and “COI”, “cox1” or “CO1”, requesting only sequences between 450 and 1,000 bp in length. Searches for longer sequences (i.e. mitochondrial genomes) did not reveal any species not already sampled. Any sequences generated from specimens collected outside of South America, or those that clearly appeared to be attributed to incorrect species names were removed.

### Single locus discovery of species

We used four single-locus species-discovery (SLSD) methods to partition our dataset into putative species-like clusters: (1) GMYC, the general mixed Yule coalescent model^33,43,61^; (2) bGMYC, a Bayesian implementation of the GMYC^48^; (3) local minima (locMin), a distance threshold optimising and clustering approach from the spider_1.3-0 software package^62^; and (4) mPTP, the poisson tree process method^44,45^. Unless otherwise stated, analyses were carried out in R 3.4.1^63^. Beast 1.8.4^64^ was used to generate a posterior sample of ultrametric trees for the GMYC analyses. The dataset was first collapsed to unique haplotypes^65^. The Beast analysis was set up as follows: substitution model TN93 + Γ as selected by jModeltest2^66^; single model partition; strict molecular clock (relaxed clock was tested for *a priori*); fixed arbitrary substitution rate of 0.01; and coalescent tree prior^33,65^. Three independent chains were run for 20 million generations from random starting topologies, and were sampled every 18,000 generations, resulting in 3,333 trees (of which 333 were discarded as burn-in). The 3,000 post burn-in trees were combined and then subsampled to 1,000 for all downstream analyses. Tracer^67^ was used to verify the chains had reached stationarity.

GMYC, bGMYC and mPTP analyses were carried out as: (1) a point estimate based on the maximum clade credibility tree created in TreeAnnotator 1.8.4 (node heights “ca”); and (2) confidence intervals calculated from the posterior sample of 1,000 trees. We used the bGMYC_1.0.2^48^, splits_1.0-19^43^ and ape_4.1^68^ packages. The bGMYC posterior samples were summarised into putative species with a conservative posterior probability of conspecificity at 0.05. For mPTP (single lambda), the Beast chronograms (ultrametric trees with branch lengths scaled by time) were first transformed into phylograms (branch lengths scaled by substitutions per site) using maximum likelihood optimisation in phangorn_2.2.0^69^ under the same substitution model settings as described above. The locMin analyses were again conducted as a point estimate, and also on a set of 1,000 bootstrapped datasets to generate a confidence interval for this method.

### Data availability

New sequence data generated here are available from the GenBank nucleotide archive under the accessions MG751915–MG752866, and at the Barcode of Life BOLD database under the project name “PRNHA”. Metadata for all sequences used in this study are presented in Supplementary Table S1 as a comma delimited flatfile following Darwin Core standard vocabulary (http://rs.tdwg.org/dwc/terms/index.htm). The datasets and scripts used in this study are available from a public GitHub repository hosted at https://github.com/legalLab/publications.

## Results

### Sampling and data description

**Figure 1:**
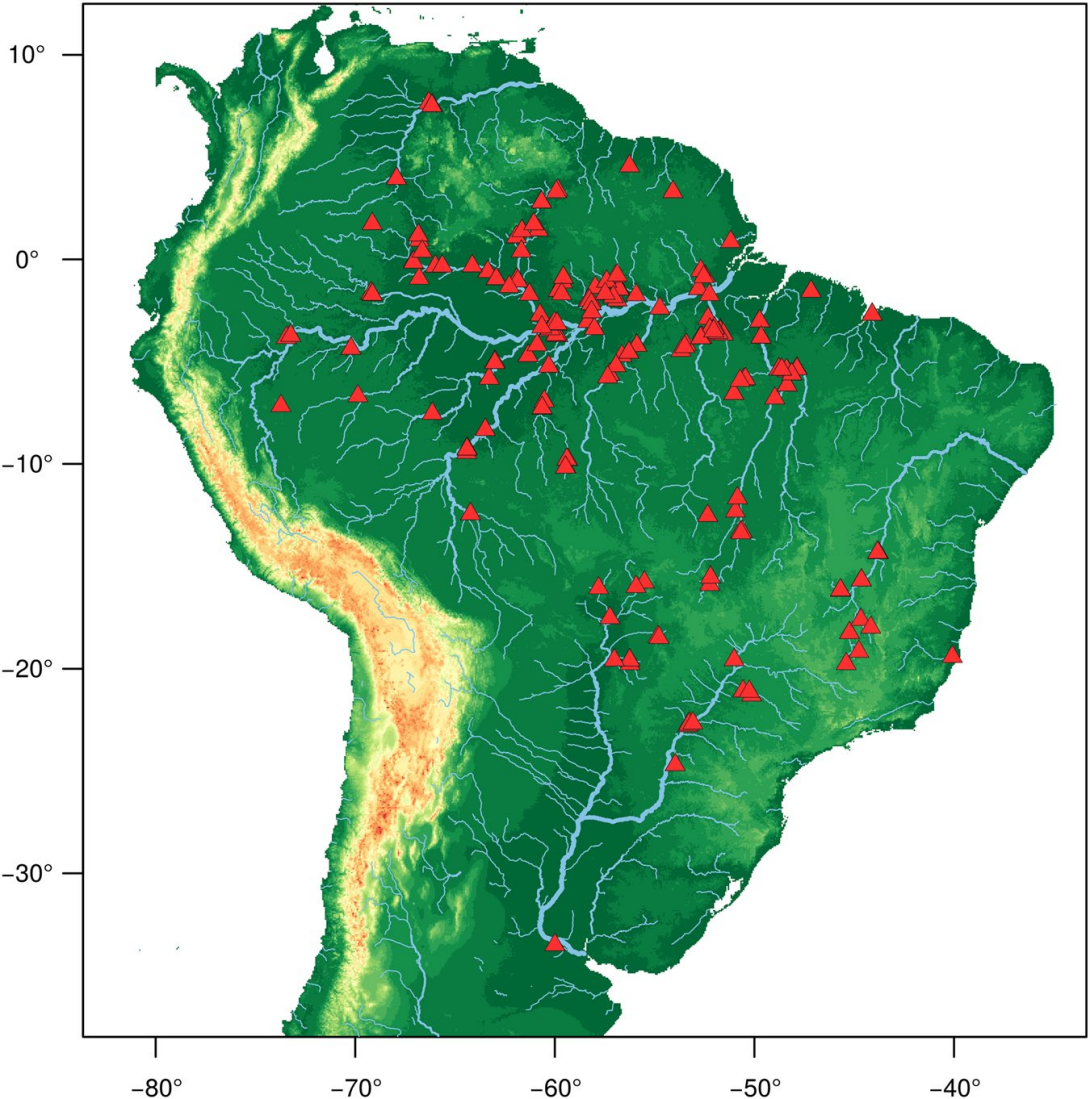
Map of unique sampling locations shown by red triangles. Of the total 208 unique sampling locations in the study, 188 with GPS coordinates are shown here. Total includes all data accessed from GenBank in addition to collections made as part of this study. Figure was created in R 3.4.1^63^ from void-filled elevation (30 sec DEM) raster and river network (RIV) shape files obtained from the World Wildlife Fund *HydroSHEDS* project^96^, and used under the following license. This product incorporates data from the *HydroSHEDS* database which is ©World Wildlife Fund, Inc. (2006–2013) and has been used herein under license. WWF has not evaluated the data as altered and incorporated within, and therefore gives no warranty regarding its accuracy, completeness, currency or suitability for any particular purpose. Portions of the *HydroSHEDS* database incorporate data which are the intellectual property rights of ©USGS (2006–2008), NASA (2000–2005), ESRI (1992–1998), CIAT (2004–2006), UNEP–WCMC (1993), WWF (2004), Commonwealth of Australia (2007), and Her Royal Majesty and the British Crown and are used under license. The *HydroSHEDS* database and more information are available at http://www.hydrosheds.org.

As part of this study a total of 975 serrasalmid individuals were collected from 168 unique localities in 30 major river drainages (Fig. 1). With the addition of data from GenBank this increased to 1,122 specimens from 208 unique localities in 34 major drainages. Upon morphological assessment a total of 68 species-level taxa were identified and we were able to assign taxonomic names to 60 of these (i.e. valid nominal species), with a further eight being identified to genus level only, i.e. putative new species (six pacus, two piranhas). Data for thirteen species were obtained from GenBank, but only one of these (*Serrasalmus marginatus*) was not already present in our dataset (bringing the total to 69 species). Overall, the sampling covered 61 (19 piranha species and 42 pacu species) of the 94 (65%) valid serrasalmid species representing all 16 genera (100%). Per species, 63 of the total 69 (91%) were represented by more than one individual, with 56 (81%) represented by five or more individuals; median number of individuals per species was ten, mean was 16.3, and maximum was 95 (*Serrasalmus rhombeus*). Fifty-one species (74%) were collected from more than one locality; 45 (65%) were collected from three or more localities; median number of localities per species was four, mean was 6.2, and maximum was 35 (*Serrasalmus rhombeus*). Forty species (58%) were collected in more than one drainage; 33 (48%) were collected from three or more drainages; median number of drainages per species was two, mean was 3.6, and maximum was 15 (*Serrasalmus rhombeus*). The aligned DNA barcode matrix comprised 1,122 taxa by 621 bp. The unaligned sequences varied in length 417–621 bp; 11 (1%) were less than 500 bp, 97% were greater than 530 bp, 37% were the full 621 bp; median sequence length was 609 bp (mean 595 bp). The dataset comprised a total of 444 unique haplotypes. Per species (Table 1), 59 (86%) were represented by more than one haplotype, with 28 (41%) represented by five or more haplotypes; median number of haplotypes per species was four, mean was six, and maximum was 29 (*Serrasalmus rhombeus*).

**Table 1:** Dataset statistics broken down per species (species assigned from morphological assessment and including valid nominal species and putatively undescribed species), including (from left to right): individual count, number of haplotypes, number of sampled locations, number of sampled major drainages, maximum intraspecific divergence (p-distance), minimum interspecific divergence (p-distance), monophyly, and number of delimited clusters by method (mPTP, locMin, bGMYC, GMYC). Totals (and mean values per species in parentheses) are given in the final row.

Species name	Individuals	Haplotypes	Drainages	Localities	Max. intra.	Min. inter.	Monophyletic	Clusters
*Acnodon normani*	2	2	1	1	0.007	0.011	True	1,1,1,1
*Acnodon senai*	2	1	1	1	0	0.011	True	1,1,1,1
*Catoprion mento*	19	10	7	10	0.016	0.094	True	1,1,2,3
*Colossoma macropomum*	30	14	3	3	0.013	0.112	True	1,1,1,1
*Metynnis* aff. *lippincottianus*	3	3	3	2	0.011	0.069	True	1,1,1,1
*Metynnis altidorsalis*	6	5	5	5	0.024	0.011	False	1,2,2,2
*Metynnis cuiaba*	13	5	4	7	0.008	0.011	True	1,1,1,1
*Metynnis fasciatus*	6	3	2	3	0.004	0.024	True	1,1,1,1
*Metynnis guaporensis*	19	8	7	10	0.01	0.02	True	1,1,1,1
*Metynnis hypsauchen*	37	8	11	17	0.006	0.024	True	1,1,1,1
*Metynnis lippincottianus*	20	4	6	12	0.006	0.045	True	1,1,1,1
*Metynnis luna*	10	7	3	5	0.044	0.02	False	3,3,3,3
*Metynnis maculatus*	13	4	2	5	0.004	0.017	True	1,1,1,1
*Metynnis melanogrammus*	10	5	3	4	0.016	0.061	True	2,1,2,2
*Metynnis mola*	6	4	1	5	0.007	0.069	True	1,1,1,1
*Metynnis polystictus*	6	4	1	2	0.007	0.045	True	1,1,1,1
*Metynnis* sp.2	13	4	2	2	0.006	0.015	True	1,1,1,1
*Mylesinus* aff. *paraschomburgkii*	1	1	1	1	0	0	Singleton	1,1,1,1
*Mylesinus paraschomburgkii*	15	4	3	3	0.026	0.047	True	3,3,3,3
*Mylesinus paucisquamatus*	5	3	2	2	0.008	0.04	True	1,1,1,1
*Myleus micans*	7	5	1	3	0.005	0.082	True	1,1,1,1
*Myleus setiger*	29	13	8	15	0.022	0.052	True	1,1,3,3
*Myloplus* aff. *rubripinnis*	26	12	4	10	0.107	0.02	False	6,6,6,6
*Myloplus arnoldi*	22	9	4	11	0.086	0.095	True	2,2,2,2
*Myloplus asterias*	69	29	8	16	0.064	0	False	5,5,7,7
*Myloplus levis*	2	1	1	1	0	0	True	1,1,1,1
*Myloplus lobatus*	34	7	5	9	0.007	0.026	True	1,1,1,1
*Myloplus lucienae*	6	1	1	4	0	0.026	True	1,1,1,1
*Myloplus planquettei*	1	1	1	1	0	0.034	Singleton	1,1,1,1
*Myloplus rhomboidalis*	13	3	3	4	0.023	0.097	True	3,3,3,3
*Myloplus rubripinnis*	30	16	5	6	0.066	0	False	2,3,3,3
*Myloplus schomburgkii*	38	17	7	13	0.117	0.078	False	4,4,4,4
*Myloplus* sp. ‘Tapajόs’	2	1	1	1	0	0	False	1,1,1,1
*Myloplus tiete*	1	1	1	1	0	0	Singleton	1,1,1,1
*Myloplus zorroi*	14	4	1	2	0.007	0	False	1,1,1,1
*Mylossoma aureum*	26	9	6	9	0.059	0.05	True	2,2,2,2
*Mylossoma duriventre*	75	26	14	29	0.072	0.05	True	3,3,4,4
*Ossubtus xinguense*	6	4	1	3	0.005	0.094	True	1,1,1,1
*Piaractus brachypomus*	38	8	9	11	0.033	0	False	2,2,2,2
*Piaractus mesopotamicus*	10	2	1	1	0.002	0	False	1,1,1,1
*Pristobrycon* sp. ‘Negro’	5	2	1	1	0.009	0	False	1,1,1,1
*Pristobrycon striolatus*	37	16	8	14	0.009	0.099	True	1,1,1,1
*Pygocentrus cariba*	7	3	1	1	0.004	0.039	True	1,1,1,1
*Pygocentrus nattereri*	55	15	13	18	0.062	0.002	False	3,2,4,4
*Pygocentrus piraya*	15	3	1	4	0.005	0.013	True	1,1,1,1
*Pygopristis denticulata*	11	2	5	6	0.002	0.104	True	1,1,1,1
*Serrasalmus altispinis*	8	3	3	6	0.004	0.003	True	1,1,1,1
*Serrasalmus brandtii*	15	3	1	8	0.005	0.028	True	1,1,1,1
*Serrasalmus compressus*	5	3	2	2	0.011	0.006	False	1,1,1,1
*Serrasalmus elongatus*	6	3	4	5	0.01	0.018	True	1,1,1,1
*Serrasalmus gibbus*	15	4	2	5	0.023	0	False	1,2,2,2
*Serrasalmus gouldingi*	30	8	7	11	0.007	0.019	True	1,1,1,1
*Serrasalmus hastatus*	5	2	1	1	0	0.003	True	1,1,1,1
*Serrasalmus maculatus*	7	6	3	5	0.042	0	False	3,3,2,3
*Serrasalmus manueli*	20	8	6	11	0.013	0.017	True	1,1,1,2
*Serrasalmus marginatus*	6	4	1	1	0.004	0.006	True	1,1,1,1
*Serrasalmus rhombeus*	95	18	15	35	0.023	0	False	1,1,4,4
*Serrasalmus serrulatus*	19	7	7	11	0.018	0.022	True	1,1,2,2
*Serrasalmus* sp. ‘Robertsoni’	1	1	1	1	0	0	Singleton	1,1,1,1
*Serrasalmus spilopleura*	23	11	6	11	0.014	0	False	1,1,1,2
*Tometes ancylorhynchus*	8	4	1	3	0.007	0.032	True	1,1,1,1
*Tometes camunani*	2	2	1	1	0.01	0	False	1,1,2,2
*Tometes kranponhah*	11	3	1	4	0.003	0.032	True	1,1,1,1
*Tometes lebaili*	3	2	1	1	0.002	0.068	True	1,1,1,1
*Tometes makue*	1	1	1	1	0	0.072	Singleton	1,1,1,1
*Tometes siderocarajensis*	6	2	2	3	0.01	0	False	1,1,1,1
*Tometes* sp. ‘Tapajόs’	15	6	1	3	0.012	0	False	1,1,1,1
*Tometes trilobatus*	1	1	1	1	0	0.014	Singleton	1,1,1,1
*Utiaritichthys longidorsalis*	5	2	1	1	0.002	0.021	True	1,1,1,1
Total species: 69	1,122 (16.3)	444 (6)	34 (3.6)	208 (6.2)	—	—	—	—

### Single locus discovery of species

**Figure 2:**
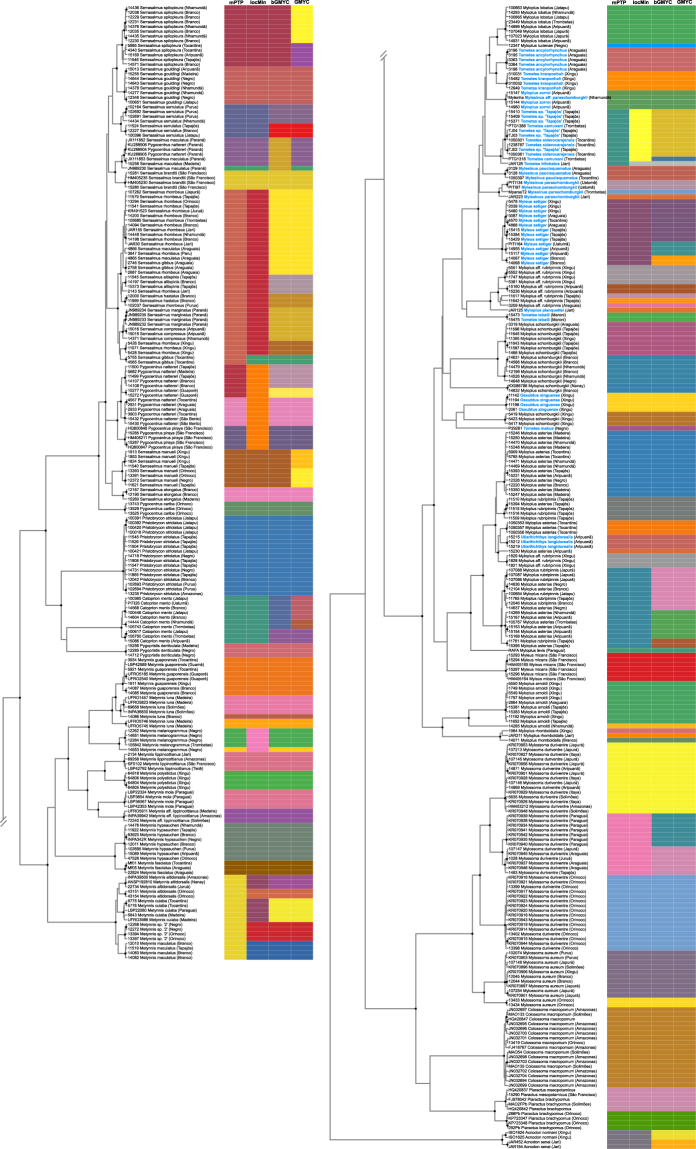
Maximum clade credibility chronogram from 1,000 posterior trees generated using Beast. Dataset comprised 444 unique haplotypes (from total 1,122) of serrasalmid COI sequences (621 aligned base pairs). Bayesian posterior probabilities above 0.95 are shown as dark nodes. Point estimate species delimitations (total 118 unique delimitations) are shown by method as coloured boxes; due the large number of unique colours, some may appear similar. Rheophilic species are highlighted in bold blue font. Tree was presented using the ggtree_1.6.11 package^97^.

Point estimates for the SLSD varied between 76 putative species (locMin) and 99 species (GMYC), with 118 unique molecular delimitations over all methods (Table 2; Fig. 2); confidence intervals (95%) were largest for locMin at 67–140 species and lowest for mPTP at 75–83 species (Table 2). Centers of the confidence interval distributions tended to be lower than the point estimates for the GMYC and bGMYC analyses, and higher for the locMin and mPTP analyses. Of the point estimate delimitations 49% were supported by congruence of all four methods, 60% were supported by three or four methods, and 14% by only one method (Fig. 2).

**Table 2:** Point estimates and confidence intervals (CIs) of numbers of species broken down by species delimitation method, with average values.

Method	Point estimate	CI 95	CI mean	CI median	CI mode
bGMYC	95	38–102	70	71	72
GMYC	99	49–118	86	88	92
locMin	78	67–140	89	86	86
mPTP	76	75–83	79	79	80

Among method point estimates, between 14 (20%; mPTP) and 23 (33%; GMYC) of the species were represented by more than one COI lineage, with between nine (13%; locMin, mPTP) and 13 (19%; GMYC) represented by three or more lineages (Table 1). Three or more lineages were reported by all methods for *Metynnis luna*, *Mylesinus paraschomburgkii*, *Myloplus asterias*, *Myloplus rhomboidalis*, *Myloplus schomburgkii*, *Myloplus* aff. *rubripinnis*, and *Mylossoma duriventre*. Of the 13 species delimited by all methods as having more than one lineage, six were within the genus *Myloplus*.

The locMin analysis optimised a divergence threshold of 0.0135 (p-distance) for the dataset. COI lineages delimited by this method varied between 0.023 maximum intraspecific divergence (*Myloplus rhomboidalis*) and 0.117 (*Myloplus schomburgkii*), while eight were greater than 0.05 (Table 1). The overall mean maximum intraspecific divergence was 0.0068 with the exclusion of the 15 species showing intraspecific genetic distances above the threshold (Table 1). Of the 69 *a priori* identified species, 43 (62%) were monophyletic, 20 (29%) were not monophyletic, and six (9%) were singletons (Table 1). Eighteen species (26%) shared haplotypes with another species, and this most commonly occurred in *Myloplus* (6 spp.) and *Serrasalmus* (5 spp.). A neighbour-joining tree showing all 1,122 COI sequences coloured by species is presented as Supplementary Fig. S1.

## Discussion

**Figure 3:**
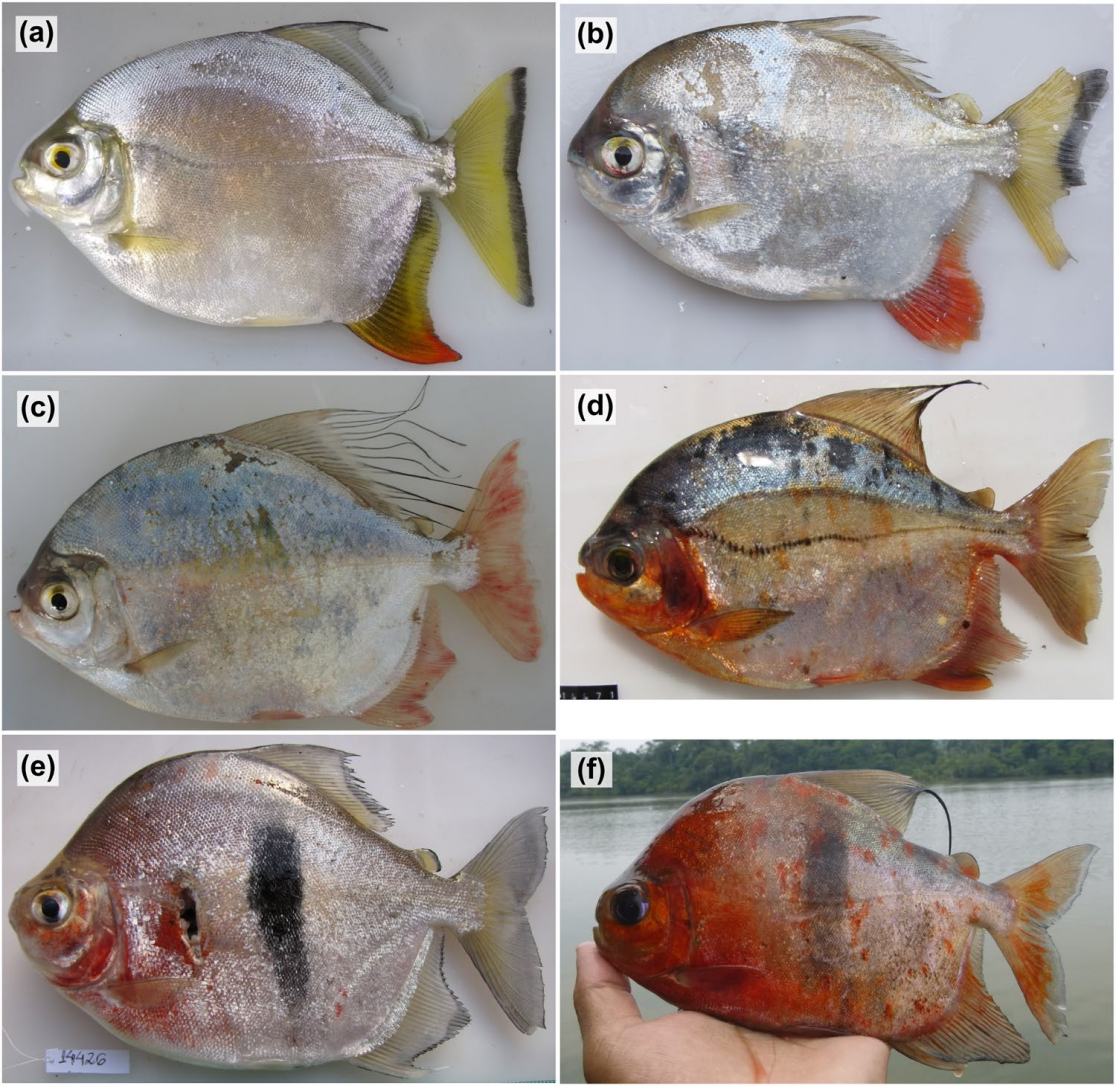
Images of freshly caught pacus highlighting groups with significant intraspecific genetic diversity: (**a**) *Myloplus arnoldi*, Tapajόs River; (**b**) *Myloplus arnoldi*, Nhamundá River; (**c**) *Myloplus asterias*, Branco River; (**d**) *Myloplus asterias*, Nhamundá River; (**e**) *Myloplus schomburgkii*, Nhamundá River; and (**f**) *Myloplus schomburgkii*, Tapajόs River. All images were taken by the authors.

In terms of variation in the pacus, of the 13 morphologically identified species of *Myloplus*, six had multiple lineages with many of these in the Guiana and Brazilian shields; in *M*. *arnoldi*, a lineage from the Guiana Shield (Nhamundá River) was identified as divergent from conspecifics in the Brazilian Shield (Araguaia, Tapajόs and Xingu rivers), separated by 0.086 p-distance (Fig. 3; Table 1); in *M*. *rhomboidalis* there were two Guiana Shield lineages (Jari and Branco rivers) and one in the Brazilian Shield (Xingu River); in *M*. *schomburgkii* (Fig. 3) there was a Guiana Shield lineage (Branco, Negro and Nhamundá rivers) distinct from a Brazilian Shield lineage (Araguaia, Tapajόs and Xingu Rivers), a third lineage found in the Xingu, as well as an intriguing forth lineage from the upper Amazon (Nanay River, Peru) and the Branco River. The species comprising *Myloplus asterias*/*rubripinnis* (including *M*. aff. *rubripinnis*) was estimated to contain between 11 and 13 lineages, with distinct lineages found in the Araguaia (one), Tocantins (one), Tapajόs (four), Aripuanã (two), Xingu (two), and Jatapu (one) rivers. Examples of within-shield diversification were apparent with two clades of multiple lineages within the Brazilian Shield, and also one lineage showing across-shield conspecificity (Aripuanã, Trombetas, Nhamundá). Extensive non-monophyly of the nominal taxa ascribed to the *Myloplus asterias*/*rubripinnis* group indicates problems in current diagnoses of the taxa and/or application of diagnostic characters supporting these taxa, and therefore given this ambiguity and the apparent high levels of within-drainage endemism, a taxonomic revision of this group should be a priority.

Other pacus also displayed large intraspecific divergences. *Mylossoma aureum*, *M*. *duriventre*, and *Piaractus brachypomus* all revealed lineages in the Orinoco distinct from those of the Amazon basin (see also Escobar *et al*.^29^ and Mateussi *et al*.^30^). *Mylesinus paraschomburgkii* showed evidence of distinct lineages in the Guiana Shield rivers (Uatumã, Trombetas and Jari), while a singleton specimen of *Mylesinus* aff. *paraschomburgkii* from the Nhamundá River was nested within *Myloplus zorroi* from the Aripuanã River. The distribution of lineages within *Myloplus lobatus* and *Myleus setiger* also indicated a biogeographic link between the Aripuanã and the Nhamundá, Jatapu, Uatumã and Trombetas rivers, reflecting the historical proximity of the mouths of these southerly flowing Guiana Shield rivers before the capture of the north flowing Brazilian Shield Aripuanã River by the Madeira River. Sharing of species and lineages between the Aripuanã and Guiana Shield rivers is not restricted to the serrasalmids, but has also been observed in cichlids of the genus *Symphysodon*^70,71^ and loricariid catfishes^72^. Aside from the three potential lineages of *Metynnis luna*, there were few new lineages of silver dollars, likely reflecting the recent taxonomic work^22^ and ongoing studies being carried out on the group (Ota, studies in progress).

Patterns among piranhas were less clear than for the pacus, with greater incongruence among methods. With 16 species considered valid, there were between 12 (locMin) and 22 (GMYC) lineages in *Serrasalmus* and *Pygocentrus*. While the distinctiveness of *Pygocentrus cariba*, *P*. *piraya*, *Serrasalmus brandtii*, *S*. *elongatus*, *S*. *gouldingi*, *S*. *manueli*, *S*. *serrulatus* and *S*. *spilopleura* were well supported by at least three of the four methods, the *Serrasalmus rhombeus* group was the primary source of incongruence. Here, the mPTP method recognised only one species in an inclusive clade comprising eight nominal taxa, and the GMYC methods recognised six species. This indicates that this group may be at the limits of resolution for a single mitochondrial locus and the methods employed here. Previous genetic analyses of the group have reported similar observations, with extensive haplotype sharing between species^73^. Regardless, several of the species within this group were recovered as monophyletic despite the low genetic divergences, including *Serrasalmus altispinis*, *S*. *compressus*, *S*. *hastatus*, and *S*. *marginatus*. Some individuals of *S*. *gibbus* and *S*. *maculatus* were nested within *S*. *rhombeus*, but these were probably the result of misidentification (Supplementary Fig. S1). Although not supported by all methods, it is possible that given the patterns observed in other taxa, the *S*. *rhombeus* individuals from the Xingu River represent a distinct species. Therefore, due to ontogenetic complexities, genetic similarity and the subtle morphological differences among species and lineages of the *S*. *rhombeus* clade, we feel it would benefit greatly from a population genomic analyses before any taxonomic treatment of the group is embarked upon. In the genus *Pygocentrus*, the species *P*. *nattereri* was found to comprise up to four lineages. One of these four was represented by GenBank samples nested within *Serrasalmus maculatus* from the Paraná River, and we believe that these samples were misidentified. Although not supported by all delimitations, the other three lineages of *P*. *nattereri* are possibly distinct, with one from the Tocantins/Araguaia/São Bento rivers and another from the Guaporé River, and both distinct from the more widespread Amazonas clade. *Serrasalmus maculatus* from the upper Paraná River was reported by three of the four methods to form a distinct lineage from *S*. *maculatus* of the lower Paraná River. The upper and lower Paraná River were distinct ichthyofaunal provinces separated by the Sete Quedas rapids until the construction of the Itaipú dam, which due to its system of locks, permitted the homogenisation of these faunas^74^.

Among the 69 *a priori* identified taxonomic species of serrasalmid analysed herein, up to 23 are represented by more than one COI lineage (Table 1). Despite recent studies and taxonomic revisions describing new species^5,21,22^, our results show that a number of potential new species may still await a formal morphological diagnosis and description. Many factors contribute to this underestimation of diversity within the family. Only few morphological studies have been published in the last 10 years, reflecting the difficulties of interpreting high levels of ontogenetic variation, allometric growth, sexual dimorphism, and spatial variation in both body shape and colour pattern^5,21,25,75,76^. The number of possibly unrecognised species observed here in serrasalmids support the conclusions of Reis *et al*.^2^, who estimated that 34–42% of Neotropical freshwater fishes remain undescribed, and are mostly concentrated in the Amazon basin. The main explanations for this unrecognised diversity stem largely from (1) historically poorly sampled areas above geological barriers such as rapids; (2) widespread taxa or heterogeneous taxa with insufficient or overwhelming amounts of museum material; or (3) cryptic or pseudocryptic (morphological differences apparent but overlooked) diversity in widespread species. Genetic data are an important instrument in uncovering cases of the latter^77^.

Of particular importance are the rheophilic taxa inhabiting rapids. Geologically the western Amazon basin is characterised by a sedimentary basin, and in the central and eastern portion by the crystalline Guiana and Brazilian Shields separated by the Amazon River^78^. As affluents of the Amazon descend the Guiana and Brazilian Shields, they form riffles, rapids and waterfalls inhabited by a distinctive fauna and flora. Aquatic flora of the rapids habitats is characterised by the Podostemaceae^79^, while perhaps the best known faunal component of these habitats are the loricariid catfishes^80^. Serrasalmids also are a conspicuous component. Our data indicate that rheophilic pacus classified in the genera *Tometes*, *Ossubtus*, *Utiaritichthys*, *Mylesinus* and *Myloplus* represent multiple, apparently evolutionarily independent lineages of rheophilic fishes (Fig. 2). Conversely, we also show lineages and haplotypes to be shared between rheophilic habitats of different rivers–e.g. the *Tometes camunani* complex–supporting the hypothesis of interconnection of these rheophilic habitats during low-water glacial periods of the Pleistocene^81^. Serrasalmids present not only a fascinating window into adaptations to extreme environments and the complexity of diversification patterns in this environment^82–84^, but the strictly rheophilic species are also the most threatened of the serrasalmids since hydroelectric projects are developed at sites of rapids and waterfalls, largely destroying these unique habitats and their associated taxa^85^.

The underestimation of the variation and diversity in the Serrasalmidae–and which can be extrapolated to Amazonian aquatic fauna in general–is directly relevant to the conservation of these groups. Due to high proportions of faunal endemism and increasing anthropogenic threats, the Brazilian Shield rivers and their faunas are of particular conservation concern^80,86,87^. Reis^88^ summarised the principal anthropogenic threats of the Amazon River basin as: extensive deforestation of Amazon forest; hydroelectric dam building with the associated transformation of lotic environments into lentic environments resulting in the extirpation or significant reduction of populations of rheophilic species (while concomitantly contributing to the proliferation of lentic-adapted species); alluvial gold mining causing mercury contamination; and overexploitation of most commercial species. Therefore, we reiterate the conclusions of Reis^88^, who suggested that one of the primary instruments for conservation of Amazon basin fishes is increasing expertise in fish taxonomy and systematics. Additionally, we advocate DNA barcoding and other genetic tools as powerful complementary methods for uncovering fish diversity and highlighting groups in need of taxonomic revisions. Here we demonstrate the utility of DNA barcoding in providing an independent estimate of species alpha diversity, and additionally in providing preliminary data on population subdivision, gene flow, and relative ages of divergences.

However, important caveats need to be considered when interpreting single locus mtDNA data. Our confidence intervals were generally wide, reflecting the influence of phylogenetic uncertainty on our results and its importance in species delimitation as a whole^48,49^. Furthermore, the failure to congruently discriminate closely related species, such as those in the *Serrasalmus rhombeus* group, is perhaps a reflection of the limitations of single threshold SLSD methods when faced with situations where species with large effective population sizes have recently diverged in rapid succession (i.e. a young radiation), conditions whereby single locus methods are known to underestimate species diversity^43,89,90^. While multiple threshold models were developed to accommodate variation in coalescent depths between groups^33,45^, we were unable to generate realistic results while experimenting with these settings (as evidenced by excessive splitting). This is largely due to multiple threshold models making possibly spurious delimitations by recognising population structuring as speciation events^91,92^. Therefore, where delimitations are implausible or incongruent we recommend secondary sources of data be (re-)examined. While rates of phenotypic evolution and speciation are correlated over macroevolutionary scale^93^, there will be situations where local adaptation and fine-scale speciation may change phenotypes at a rate significantly faster than can be identified by neutral loci^94^, emphasising the need for additional data from morphology, behaviour, distribution, and ecology^35–38^ when undertaking systematic revisions.

Geographic scale is another important factor in determining the structure of DNA barcode datasets; Bergsten *et al*.^40^ demonstrated the substantial increase in intraspecific diversity and the decrease in interspecific divergence over increasing geographical distances. Fortunately, GMYC methods have been shown to be robust to the presence of singletons and absences of intermediate haplotypes^65,89^, but where we report putatively new lineages based on low numbers of individuals, effort still needs to be made to source more specimens before more conclusive statements can be made about the distinctiveness of those taxa^95^. Overall our sampling generated a geographically broad dataset with three quarters of the species having been collected from more than one locality, and over half being collected from more than one major drainage. Despite the positive bias for samples from the eastern and central Brazilian Amazon, and the paucity of samples from the western Amazon, we are confident in having captured a significant proportion of serrasalmid diversity. Inventories from Peru, Colombia and Ecuador would be extremely valuable additions, however.

Our single locus species delimitation results support a notion that piranha and pacu taxonomic diversity is currently underestimated in the Brazilian Amazon. The four methods achieved a high level of congruence (60% of the lineages were supported by three or more methods), indicating they were recognising a common signal of diversification, with great majority of these lineages also supported as allopatric and biogeographically distinct populations. The results particularly highlight: (1) the Guiana and Brazilian Shields as regions of underestimated but high ichthyofaunal endemism and diversity; (2) the existence of both between-shield (e.g. *Myloplus schomburgkii*, *M*. *arnoldi*), and within-shield (*Myleus setiger*, *Mylesinus paraschomburgkii*, *Myloplus rubripinnis*/*asterias*) diversification patterns in pacus; (3) very recent biogeographic connection between the the Aripuanã (Brazilian Shield) and Guiana Shield rivers; (4) distinct lineages of species shared between the Amazon and Orinoco basins (*Mylossoma aureum*, *M*. *duriventre*, *Piaractus brachypomus*); (5) the evolutionary uniqueness, distinctness, and apparent independent evolution of rheophilic lineages; and (6) the taxonomic difficulties associated with piranhas. Thus, characterisation of these faunas by traditional taxonomic methods combined with further effort in sequencing more loci is needed to better understand the implications of these results in an explicit and testable biogeographic framework of Neotropical diversification and community assemblage.

## Electronic supplementary material

NJ Tree

Supplementary Dataset 1
