# Supplementary material for: One thousand DNA barcodes of piranhas and pacus reveal geographic structure and unrecognised diversity in the Amazon
Source: Sci Rep. 2018 May 30;8:8387. doi: 10.1038/s41598-018-26550-x (PMC5976771; doi:10.1038/s41598-018-26550-x)

One thousand DNA barcodes of piranhas and pacus reveal geographic structure and unrecognised diversity in the Amazon

Valeria N. Machado, Rupert A. Collins, Rafaela P. Ota, Marcelo C. Andrade, Izeni P. Farias, Tomas Hrbek

Figure S1. Neighbour joining phylogram showing all 1,122 COI sequences of serrasalmids (621 aligned basepairs), constructed from p-distances. Individuals are coloured by taxonomic name to highlight incongruences.

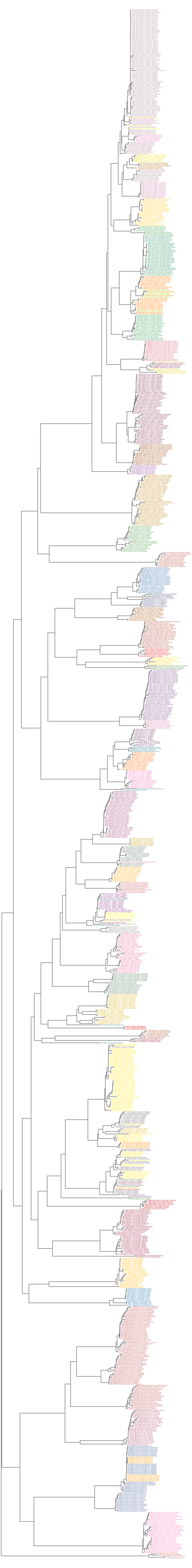

Supplement: Supplementary file 1 — NJ Tree [file 41598_2018_26550_MOESM1_ESM.pdf]
